# Neighborhood Vulnerability and Age of Natural Menopause and Menopausal Symptoms Among Midlife Women

**DOI:** 10.1001/jamanetworkopen.2025.12075

**Published:** 2025-05-22

**Authors:** Zhi Lin, Sheryl L. Rifas-Shiman, Wei Perng, Melissa Pérez Capotosto, Jan L. Shifren, Hadine Joffe, Marie-France Hivert, Jorge E. Chavarro, Emily Oken, Izzuddin M. Aris

**Affiliations:** 1 Harvard Medical School, Boston, Massachusetts; 2 Department of Population Medicine, Harvard Medical School and Harvard Pilgrim Health Care Institute, Boston, Massachusetts; 3 Lifecourse Epidemiology of Adiposity and Diabetes Center, University of Colorado Anschutz Medical Campus, Aurora; 4 Department of Epidemiology, University of Colorado Anschutz Medical Campus, Aurora; 5 W.F. Connell School of Nursing, Boston College, Boston, Massachusetts; 6 Department of Obstetrics, Gynecology, and Reproductive Biology, Massachusetts General Hospital, Boston; 7 Mary Horrigan Connors Center for Women’s Health and Gender Biology, Brigham and Women’s Hospital, Boston, Massachusetts; 8 Diabetes Unit, Massachusetts General Hospital, Boston; 9 Department of Nutrition, Harvard T.H. Chan School of Public Health, Boston, Massachusetts

## Abstract

**Importance:**

Women experiencing more severe menopausal symptoms exhibit poorer quality of life, and those with early menopause have a higher risk of developing chronic diseases. However, the extent to which neighborhood disadvantage contributes to menopause onset and symptom severity remains understudied.

**Objective:**

To examine the association of Social Vulnerability Index (SVI) with age of natural menopause onset and menopausal symptom severity.

**Design, Setting, and Participants:**

This cohort study used data from a prospective cohort of women participating in Project Viva who were initially enrolled in eastern Massachusetts and followed up from pregnancy to midlife between April 1999 and August 2021. Participant inclusion required geocoded residential addresses at enrollment (1999-2002), 8-year follow-up (2006-2010), and 13-year follow-up (2012-2016); age at natural menopause; and menopausal symptoms. Data were analyzed between March 1 and June 30, 2024.

**Exposures:**

SVI grouped into 5 categories: very low (<20th percentile), low (20th to <40th percentile), moderate (40th to <60th percentile), high (60th to <80th percentile), or very high (≥80th percentile) vulnerability.

**Main Outcomes and Measures:**

Age at natural menopause and self-reported menopausal symptoms based on the presence and severity of 11 symptoms over the past year. These symptoms were assessed using the Menopause Rating Scale (total score range: 0-44, with higher scores indicating greater severity).

**Results:**

Of the 691 women included in the study (mean [SD] enrollment age, 33.7 [3.8] years; 41 with Asian [6.0%], 79 with Black [11.5%], 39 with Hispanic [5.7%], 507 with White [73.6%], and 23 with other [3.3%] race and ethnicity), 87 (12.6%) resided in neighborhoods with very high SVI at enrollment, 38 of 635 (6.0%) at 8-year follow-up, and 41 of 660 (6.2%) at 13-year follow-up. The Kaplan-Meier estimate for median age of natural menopause was earlier in women residing in neighborhoods with very high vs very low SVI at enrollment (52.0 [95% CI, 51.0-53.0] years vs 53.0 [95% CI, 53.0-54.0] years), 8-year follow-up (51.0 [95% CI, 50.0-53.0] years vs 53.0 [95% CI, 53.0-54.0] years), and 13-year follow-up (51.0 [95% CI, 50.0-53.0] years vs 53.0 [95% CI, 53.0-54.0] years). After adjusting for covariates, residence in neighborhoods with very high (but not low, moderate, or high) vs very low SVI at enrollment (adjusted hazard ratio [AHR], 1.36; 95% CI, 0.90-2.06), 8-year follow-up (AHR, 2.23 (95% CI, 1.29-3.85), and 13-year follow-up (AHR, 2.18 (95% CI, 1.30-3.66) was associated with higher risk of earlier natural menopause. SVI was not associated with menopausal symptoms.

**Conclusions and Relevance:**

In this cohort study, women who resided in neighborhoods with very high vulnerability within 10 years of the perimenopause period exhibited higher risk of earlier natural menopause. Future research is warranted to explore whether initiatives to improve neighborhood conditions could mitigate the association of neighborhood disadvantage with earlier menopause onset.

## Key Points

**Question:**

Are there associations between neighborhood-level measures of social vulnerability and age of natural menopause and menopausal symptoms?

**Findings:**

In this cohort study of 691 parous women followed up from pregnancy to midlife, those residing in neighborhoods with very high vulnerability exhibited higher risk of earlier natural menopause onset by approximately 2 years compared with women in neighborhoods with very low vulnerability. No association between neighborhood vulnerability and menopausal symptom severity was observed.

**Meaning:**

The findings suggest the need for future studies examining whether strategies that address disparate contexts within neighborhoods would mitigate the association of neighborhood vulnerability with early menopause and help achieve more equitable reproductive health outcomes among high-risk women.

## Teaser

This cohort study examines the association of the neighborhood environment with the onset and severity of menopause at midlife.

## Introduction

During midlife, women transition through menopause, a period marked by major changes in female reproductive hormones often accompanied by physical and psychological symptoms.^1^ Women experiencing more severe menopausal symptoms (eg, hot flashes, night sweats, and insomnia) have exhibited poorer quality of life^2,3^ and lower cognitive performance.^4^ Furthermore, those with early menopause (aged younger than 45 years) have shown higher risks of developing chronic diseases.^5,6^ While related, age of menopause onset and menopausal symptom severity are distinct facets of reproductive aging with different underlying physiological processes.^7,8^ Identifying risk factors associated with these conditions is of clinical and public health importance and could guide strategies to prevent or mitigate these adverse health outcomes.

Prior studies have shown that individual-level factors (eg, lifestyle behaviors and psychosocial stressors) are associated with earlier age of natural menopause and higher menopausal symptom severity.^9,10,11,12,13,14,15^ These factors may themselves be perpetuated by living in disadvantaged neighborhoods, which are typically characterized by unfavorable physical and social attributes (eg, reduced greenspace, increased residential air pollution, and lack of social networks) that could exacerbate underlying stressors and worsen health outcomes. Substantial gaps remain in our understanding of how the neighborhood, an important social determinant of health,^16^ is associated with reproductive aging outcomes in women during midlife. Cohort studies in Asia^17^ and Europe^18^ have reported the associations of specific neighborhood indicators (ie, less greenspace and more residential air pollution) with earlier age at menopause. These studies, however, were conducted outside of the US and examined only singular physical neighborhood characteristics, which do not capture the totality of the physical and social experiences in the neighborhood environment. The Social Vulnerability Index (SVI)^19^—a relative measure of neighborhood disadvantage—has been increasingly used by researchers to understand the role of neighborhood vulnerability in shaping adverse health outcomes in children^20,21^ and adults.^22^ However, it remains unclear whether neighborhood vulnerability during pregnancy and perimenopause is differentially associated with age of natural menopause and symptom severity. Understanding these relationships could facilitate better targeting of preventive efforts to high-risk women during pregnancy and menopause.

To address these research gaps, we used data from a longitudinal cohort of women to examine the associations of SVI with age of natural menopause onset and menopausal symptom severity. We hypothesized that women residing in neighborhoods with higher social vulnerability during pregnancy and/or perimenopause would experience earlier menopause and more severe menopausal symptoms.

## Methods

### Study Population

We analyzed data from participants in Project Viva, an ongoing prospective cohort study of prenatal and perinatal factors in maternal, fetal, and child health, who were initially enrolled in eastern Massachusetts and followed up from pregnancy to midlife between April 1999 and August 2021. Project Viva recruited pregnant women attending their first prenatal visit between 1999 and 2002 (median [IQR] gestation time, 9.9 [8.7-11.3] weeks), and in-person follow-up visits occurred at 8 years (2006-2010), 13 years (2012-2016), and 18 years (midlife visit; 2017-2021) after enrollment. All participants provided written informed consent at enrollment and follow-up visits. The Harvard Pilgrim Health Care Institutional Review Board approved this cohort study. We followed the Strengthening the Reporting of Observational Studies in Epidemiology (STROBE) reporting guideline.

Details on recruitment and eligibility criteria for Project Viva are described elsewhere.^23,24^ Briefly, of 2100 women with singleton births, 1553 had not disenrolled prior to the midlife visit, of whom 775 participated. To restrict the analytic sample to women who were potentially undergoing natural menopausal transition, we excluded those younger than 45 years who had not experienced menopause by the time of the last administered questionnaire after the midlife visit. Prior studies have indicated that endocrine changes characteristic of the natural onset of perimenopause begin around age 45 years, and few women reach perimenopause naturally before this age.^25^ The final sample included participants with data on residential addresses at enrollment, 8-year follow-up visit, and 13-year follow-up visit and with menopausal symptoms.

### SVI

The Centers for Disease Control and Prevention developed and validated the SVI to identify populations with high risk for adverse health outcomes during public health emergencies.^19^ The SVI is derived from 15 US Census variables grouped into 4 domains: socioeconomic status, household composition and disability, racial and ethnic minority status, and housing and transportation type (eTable 1 in Supplement 1). Using geospatial software (ArcGIS; Esri), we geocoded each participant’s residential address obtained at enrollment (1999-2002), 8-year follow-up visit (2006-2010), and 13-year follow-up visit (2012-2016); we chose these visits as they were closest in time to SVI data in 2000, 2010, and 2016. We assigned a Census tract location to each address using the 2000 (for addresses at enrollment) or 2010 (for addresses at 8-year and 13-year follow-up) US Census tract boundaries, and we linked the locations to SVI data. In accordance with prior studies,^20,21^ we grouped Census tracts into the following categories based on nationwide distribution of the SVI: very low (<20th percentile), low (20th to <40th percentile), moderate (40th to <60th percentile), high (60th to <80th percentile), or very high (≥80th percentile) vulnerability.

### Menopausal Outcomes

At the midlife visit (median [IQR] age, 51.6 [49.2-54.8] years), participants self-reported the presence and severity of 11 different menopausal symptoms over the past year, which were assessed using the Menopause Rating Scale (MRS).^26^ The MRS evaluates somatic, psychological, and urogenital domains of menopausal symptoms. Each symptom was scored from none (0 points) to very severe (4 points), which was summed for a total score of 0 to 44 points. This validated approach has been shown to have high internal consistency and high test-retest reliability.^26^ We included women who completed at least 10 of the 11 items and calculated the total and domain-specific MRS scores. Participants also reported information on menopausal status annually using questionnaires, beginning at the 13-year follow-up visit until approximately 2 years after the midlife visit and, for those who had reached this milestone, age at menopause. Specifically, participants reported whether their menstrual periods had stopped for at least 12 months and, if so, the natural or secondary reason (ie, surgical, radiation, or chemotherapy).

### Covariates

At enrollment, we collected self-reported sociodemographic and clinical information through in-person interviews and medical records, which we operationalized as follows: age, race and ethnicity (Hispanic, non-Hispanic Asian [hereafter Asian], non-Hispanic Black [hereafter Black], non-Hispanic White [hereafter White], or non-Hispanic other [American Indian or Alaska Native, Native Hawaiian or Other Pacific Islander, or unspecified other race]), educational level (college graduate or non–college graduate), employment status (employed or unemployed), annual household income (≤$70 000 or >$70 000 per year), smoking status (never smoked, former smoker, or smoked during pregnancy), number of cigarettes smoked per day, and prepregnancy weight and height from which we calculated prepregnancy body mass index (BMI; calculated as weight in kilograms divided by height in meters squared). We also obtained information on employment status at the 1-year and 8-year follow-up visits as well as annual household income, smoking status, number of cigarettes smoked per day, and BMI at the 8-year and 13-year follow-up visits. We assessed race and ethnicity because we considered these variables to be proxy measures of structural racism, which has implications for residence in disadvantaged neighborhoods and other factors possibly associated with menopause outcomes. We selected these covariates based on previous publications examining associations between neighborhood environment and health outcomes in women.^17,18,27,28,29,30^

### Statistical Analysis

We analyzed correlations between SVI categories at different follow-up periods using the Spearman correlation. We used the Kaplan-Meier method to estimate the median age at natural menopause onset according to SVI categories at enrollment, 8-year follow-up, and 13-year follow-up. We fitted 3 separate Cox proportional hazards regression models to investigate associations of SVI at enrollment, 8-year follow-up, or 13-year follow-up, respectively, with age of natural menopause onset; the proportional hazards assumption was satisfied for each model. These models enabled examination of time to natural menopause onset and ensured that all participants in the analytic sample contributed to the analysis up through their last observation times.^31^ We used age as the time scale and occurrence of natural menopause as the event of interest. The hazard ratio (HR) can be interpreted as the risk of natural menopause onset within the next year at any point during follow-up. Thus, compared with those in the reference SVI category (ie, very low vulnerability), an HR higher than 1 would indicate that those residing in neighborhoods with higher SVI have an earlier age of natural menopause, whereas an HR lower than 1 would indicate a later age of menopause. We censored participants who had not experienced menopause by the time of the last administered questionnaire after the midlife visit at the age reported on the questionnaire. We also censored those who experienced menopause secondary to surgery, radiotherapy, or chemotherapy (which is biologically different from natural menopause onset^32^) at the age of secondary menopause. We used linear regression models to estimate the association of SVI at enrollment, 8-year follow-up, or 13-year follow-up with total and domain-specific MRS scores.

We adjusted all models for covariates obtained at enrollment; for models with SVI at the 8-year or 13-year follow-up visits, we also adjusted for covariates contemporaneous with the SVI exposure. We did not adjust for race and ethnicity because we viewed them as societal constructs rather than deterministic biological causes of disease risk.^33,34,35,36^ In all models, we included Census tract–level random intercepts to account for clustering of women residing in the same neighborhood. We used chained equation multiple imputation to impute values for missing covariates. We generated 25 imputed datasets for all 2100 participants. The imputation model included SVI, menopause outcomes, and covariates under study. We combined imputed datasets using the Rubin rules after excluding the women who did not satisfy the inclusion criteria. We also conducted several secondary analyses to test the robustness of the associations (eMethods in Supplement 1).

We performed all analyses between March 1 and June 30, 2024, using SAS (SAS Institute Inc). When interpreting findings, we focused on the direction, strength, and precision of the estimates. A 2-sided α < .05 was used for assessment of statistical significance.

## Results

The 691 women in the final sample had a mean (SD) age at enrollment of 33.7 (3.8) years, of whom 41 identified as Asian (6.0%), 79 as Black (11.5%), 39 as Hispanic (5.7%), 507 as White (73.6%), and 23 as other (3.3%) race and ethnicity. Overall, 313 women (46.0%) reached natural menopause by the end of the follow-up period (Table 1). The proportion of women living in neighborhoods with very high vulnerability was 12.6% (87 of 691) at enrollment, 6.0% (38 of 635) at 8-year follow-up, and 6.2% (41 of 660) at 13-year follow-up (eFigure 1 in Supplement 1). There was a moderate to strong correlation between SVI categories at enrollment and 8-year follow-up (ρ = 0.51; *P* < .001), at enrollment and 13-year follow-up (ρ = 0.43; *P* < .001), and at 8-year and 13-year follow-up (ρ = 0.74; *P* < .001). Participants residing in neighborhoods with very high vulnerability at enrollment, 8-year follow-up, or 13-year follow-up were less likely to have annual household incomes higher than $70 000 per year and more likely to have smoked during pregnancy and have higher prepregnancy BMI compared with participants in neighborhoods with very low SVI (Table 1; eTable 2 in Supplement 1). Compared with women included in the analysis, those excluded were less likely to earn annual household incomes higher than $70 000 per year, to have college degrees, and to be employed and were more likely to reside in neighborhoods with very high vulnerability at enrollment (eTable 3 in Supplement 1).

**Table 1.** Characteristics of Study Participants

Characteristic	Participants by SVI categories, No. (%)						*P* value
	Overall (N = 691)	Very low (n = 240 [34.7%])	Low (n = 145 [21.0%])	Moderate (n = 142 [20.5%])	High (n = 69 [10.0%])	Very high (n = 87 [12.6%])	
**At enrollment**							
Age, mean (SD), y	33.7 (3.8)	34.4 (3.5)	33.3 (3.9)	33.4 (3.7)	33.2 (3.6)	33.3 (4.1)	.01
Annual household income >$70 000/y	443 (68.3)	194 (84.3)	107 (75.9)	88 (65.2)	21 (36.2)	27 (35.1)	<.001
College graduate	550 (79.8)	213 (88.8)	118 (81.9)	117 (82.4)	50 (73.5)	46 (52.9)	<.001
Employed	564 (87.6)	204 (89.5)	122 (87.1)	121 (88.3)	49 (83.1)	60 (83.3)	.54
Smoking status							
Never	498 (72.3)	158 (66.4)	100 (69.0)	110 (77.5)	57 (82.6)	68 (78.2)	.01
Former	137 (19.9)	61 (25.6)	34 (23.4)	23 (16.2)	8 (11.6)	8 (9.2)	
Smoker during pregnancy	54 (7.8)	19 (8.0)	11 (7.6)	9 (6.3)	4 (5.8)	11 (12.6)	
No. of cigarettes smoked per day, mean (SD)	0.2 (0.9)	0.1 (0.7)	0.2 (1.1)	0.1 (0.8)	0.1 (0.2)	0.3 (1.5)	.28
Race and ethnicity^a^							
Hispanic	39 (5.7)	4 (1.7)	6 (4.2)	8 (5.6)	9 (13.2)	12 (13.8)	<.001
Non-Hispanic Asian	41 (6.0)	8 (3.3)	11 (7.6)	8 (5.6)	8 (11.8)	6 (6.9)	
Non-Hispanic Black	79 (11.5)	6 (2.5)	3 (2.1)	10 (7.0)	14 (20.6)	44 (50.6)	
Non-Hispanic White	507 (73.6)	218 (90.8)	121 (84.0)	109 (76.8)	35 (51.5)	19 (21.8)	
Non-Hispanic other^b^	23 (3.3)	4 (1.7)	3 (2.1)	7 (4.9)	2 (2.9)	6 (6.9)	
Prepregnancy BMI, mean (SD)	24.5 (5.1)	24.0 (4.4)	24.3 (4.8)	24.6 (5.3)	24.3 (4.8)	26.4 (6.4)	.004
**Midlife outcomes**							
Total MRS score, mean (SD)	7.9 (5.8)	7.9 (5.3)	7.4 (5.3)	8.3 (6.4)	8.2 (5.6)	7.8 (6.4)	.74
Psychological domain	2.9 (2.7)	2.7 (2.5)	2.9 (2.7)	3.1 (2.9)	3.0 (2.8)	2.7 (2.6)	.69
Somatic domain	3.4 (2.5)	3.4 (2.2)	3.2 (2.2)	3.4 (2.9)	3.3 (2.2)	3.7 (2.8)	.58
Urogenital domain	1.7 (2.0)	1.8 (2.0)	1.4 (1.8)	1.8 (1.9)	1.9 (2.1)	1.4 (2.2)	.08
Periods stopped naturally	313 (46.0)	117 (49.8)	62 (42.8)	61 (43.9)	31 (46.3)	38 (44.2)	.67

Abbreviations: BMI, body mass index (calculated as weight in kilograms divided by height in meters squared); MRS, Menopause Rating Scale (total score range: 0-44, with higher scores indicating greater severity); SVI, Social Vulnerability Index.

^a^ Self-reported.

^b^ Included American Indian or Alaska Native, Native Hawaiian or Other Pacific Islander, or unspecified other race.

The Kaplan-Meier estimate for median age of natural menopause was earlier among participants residing in neighborhoods with very high vs very low vulnerability at enrollment (52.0 [95% CI, 51.0-53.0] years vs 53.0 [95% CI, 53.0-54.0] years), 8-year follow-up (51.0 [95% CI, 50.0-53.0] years vs 53.0 [95% CI, 53.0-54.0] years), and 13-year follow-up (51.0 [95% CI, 50.0-53.0] years vs 53.0 [95% CI, 53.0-54.0] years) (Table 2). After adjusting for covariates, the adjusted HR for risk of earlier natural menopause among participants residing in neighborhoods with very high (vs very low) vulnerability was 1.36 (95% CI, 0.90-2.06) at enrollment, 2.23 (95% CI, 1.29-3.85) at 8-year follow-up, and 2.18 (95% CI, 1.30-3.66) at 13-year follow-up. However, residence in neighborhoods with low, moderate, or high vulnerability was not associated with menopause onset (Figure). SVI at all time points was not associated with total or domain-specific MRS scores (Table 3).

**Table 2.** Kaplan-Meier Estimates for Median Age of Natural Menopause Onset According to SVI Categories at Enrollment and Follow-Up

SVI categories	Estimated median age of natural menopause onset (95% CI), y		
	Enrollment (N = 691)	8-y Follow-up (n = 635)	13-y Follow-up (n = 660)
Very low vulnerability	53.0 (53.0-54.0)	53.0 (53.0-54.0)	53.0 (53.0-54.0)
Low vulnerability	53.0 (52.0-54.0)	53.0 (53.0-54.0)	53.0 (52.0-54.0)
Moderate vulnerability	53.0 (52.0-54.0)	53.0 (52.0-54.0)	53.0 (52.0-55.0)
High vulnerability	52.0 (51.0-55.0)	51.0 (50.0-54.0)	53.0 (51.0-53.0)
Very high vulnerability	52.0 (51.0-53.0)	51.0 (50.0-53.0)	51.0 (50.0-53.0)
Log-rank *P* value	.12	.02	.001

Abbreviation: SVI, Social Vulnerability Index.

**Figure.**
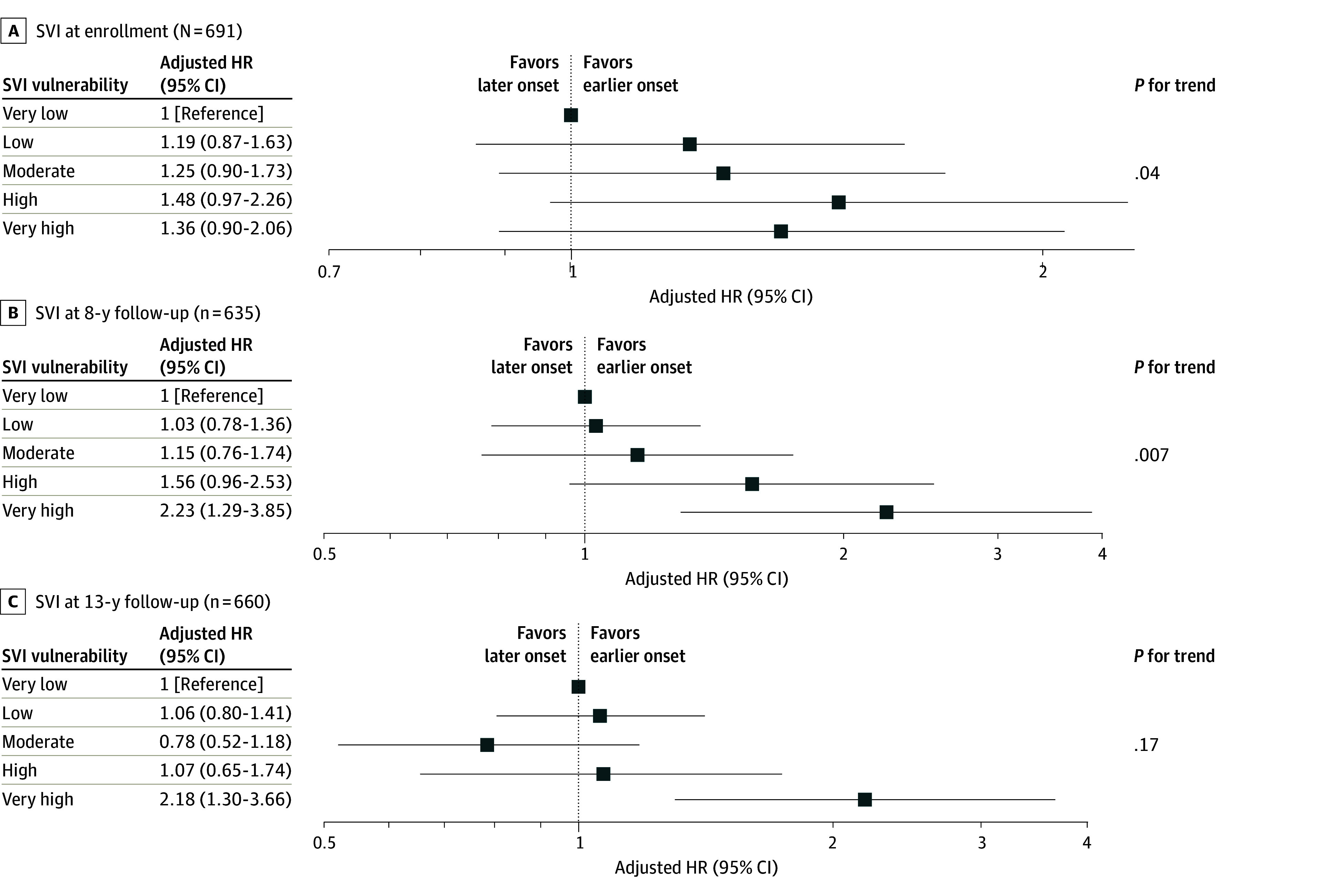
Associations of Social Vulnerability Index (SVI) at Enrollment, 8-Year Follow-Up, and 13-Year Follow-Up With Age of Natural Menopause Onset Estimates for SVI at enrollment and at 8- and 13-year follow-up were adjusted for covariates at enrollment (age, educational level, annual household income, smoking status, and prepregnancy body mass index [BMI]). Additionally, estimates for SVI at 8-year follow-up were adjusted for covariates at 1-year (employment status) and 8-year follow-up (annual household income, smoking status, number of cigarettes smoked per day, and BMI). Estimates for SVI at 13-year follow-up were also adjusted for covariates at 13-year follow-up (employment status, annual household income, smoking status, number of cigarettes smoked per day, and BMI). HR indicates hazard ratio.

**Table 3.** Associations of SVI With Total and Domain-Specific Menopausal Symptoms Score

	Effect estimate (95% CI)			
	Total score	Domain score		
		Psychological	Somatic	Urogenital
**SVI at enrollment (n = 679)^a^**				
Very low vulnerability	1 [Reference]	1 [Reference]	1 [Reference]	1 [Reference]
Low vulnerability	−0.32 (−1.50 to 0.87)	0.21 (−0.35 to 0.76)	−0.20 (−0.71 to 0.31)	−0.32 (−0.73 to 0.08)
Moderate vulnerability	0.45 (−0.76 to 1.66)	0.37 (−0.19 to 0.94)	−0.02 (−0.54 to 0.49)	0.10 (−0.31 to 0.52)
High vulnerability	0.10 (−1.52 to 1.71)	0.19 (−0.56 to 0.95)	−0.35 (−1.04 to 0.34)	0.25 (−0.30 to 0.81)
Very high vulnerability	−0.55 (−2.10 to 1.01)	−0.13 (−0.86 to 0.59)	−0.04 (−0.70 to 0.63)	−0.37 (−0.91 to 0.16)
**SVI at 8-y follow-up (n = 632)^b^**				
Very low vulnerability	1 [Reference]	1 [Reference]	1 [Reference]	1 [Reference]
Low vulnerability	0.52 (−0.54 to 1.58)	0.34 (−0.15 to 0.82)	0.10 (−0.36 to 0.55)	0.09 (−0.28 to 0.46)
Moderate vulnerability	0.45 (−1.05 to 1.95)	−0.03 (−0.72 to 0.67)	0.52 (−0.13 to 1.16)	−0.04 (−0.56 to 0.48)
High vulnerability	−0.94 (−2.81 to 0.93)	−0.45 (−1.31 to 0.41)	−0.21 (−1.02 to 0.59)	−0.28 (−0.93 to 0.37)
Very high vulnerability	0.48 (−1.67 to 2.62)	−0.21 (−1.19 to 0.78)	0.66 (−0.26 to 1.58)	0.02 (−0.72 to 0.77)
**SVI at 13-y follow-up (n = 656)^c^**				
Very low vulnerability	1 [Reference]	1 [Reference]	1 [Reference]	1 [Reference]
Low vulnerability	0.29 (−0.77 to 1.34)	0.20 (−0.29 to 0.69)	0.08 (−0.37 to 0.53)	0.01 (−0.35 to 0.37)
Moderate vulnerability	0.11 (−1.33 to 1.55)	0.34 (−0.33 to 1.01)	−0.03 (−0.64 to 0.59)	−0.20 (−0.70 to 0.30)
High vulnerability	−0.93 (−2.76 to 0.89)	−0.18 (−1.03 to 0.67)	−0.09 (−0.87 to 0.69)	−0.67 (−1.30 to 0.03)
Very high vulnerability	−0.43 (−2.46 to 1.59)	−0.29 (−1.23 to 0.66)	0.06 (−0.81 to 0.93)	−0.21 (−0.91 to 0.49)

Abbreviation: SVI, Social Vulnerability Index.

^a^ Adjusted for covariates at enrollment (age, educational level, annual household income, employment status, smoking status, number of cigarettes smoked per day, and prepregnancy body mass index [BMI]).

^b^ Adjusted for covariates at enrollment (age, educational level, annual household income, smoking status, and prepregnancy BMI), 1-year (employment status), and 8-year follow-up (annual household income, smoking status, number of cigarettes smoked per day, and BMI).

^c^ Adjusted for covariates at enrollment (age, educational level, annual household income, smoking status, and prepregnancy BMI) and 13-year follow-up (annual household income, employment status, smoking status, number of cigarettes smoked per day, and BMI).

In secondary analyses (eResults in Supplement 1), results from additional adjustment for race and ethnicity as well as inverse probability of censoring weighting analyses (to control for potential selection bias) were similar to the findings in the main analyses. The association of SVI at 8-year follow-up (but not at 13-year follow-up) with age of natural menopause did not change substantively after adjusting for SVI at previous visits (eTable 4 in Supplement 1). Additionally, while all 4 SVI subdomains contributed to the association between very high SVI and earlier menopause at 8-year follow-up, the association at 13-year follow-up was attributed primarily to the SVI subdomains of socioeconomic and household composition (eFigures 2-5 in Supplement 1).

## Discussion

In this cohort study of parous women followed up from pregnancy to midlife, we found that those residing in neighborhoods with very high (but not low, moderate, or high) compared with very low, vulnerability exhibited a higher risk of earlier natural menopause onset by approximately 2 years independent of individual sociodemographic characteristics and established factors of menopause onset, such as cigarette smoking and BMI. To put these findings in perspective, prior studies showed that smoking was associated with earlier menopause by 1.5 to 1.7 years^37,38^ and lower BMI was associated with earlier menopause by 0.4 to 0.7 years.^39,40,41^ The clinical implications of earlier age at menopause are well known; a 1-year decrease in menopause age has been associated with 2% to 3% higher risks of coronary heart disease,^42^ stroke,^43^ and mortality.^44^ These findings suggest that residence in less disadvantaged neighborhoods might be an important resilience factor that may help prevent earlier menopause onset and potentially mitigate future disease risk.

These results align with findings of prior studies that have used neighborhood metrics to illustrate associations between disadvantaged neighborhood environments and earlier age at menopause. Specifically, Guo et al^17^ reported that long-term exposure to residential air pollution was associated with earlier menopause among Asian women. Similarly, Triebner et al^18^ showed that European women residing in neighborhoods with less greenspace exhibited earlier age at menopause. However, these studies used only singular neighborhood indicators, which do not capture the complex interplay between various social and physical attributes of the neighborhood environment. These studies also did not explore the associations of neighborhood factors during pregnancy and perimenopause, which are important to examine given prior research showing differential susceptibility to social stressors at different reproductive life stages.^45^ By using a comprehensive and integrated neighborhood metric and examining the role of neighborhood vulnerability during key reproductive transitions, this study directly addresses research gaps and contributes to extant literature on neighborhoods and reproductive aging outcomes in women.

Our observation that adjustment for SVI at previous visits attenuated the association of neighborhood vulnerability with earlier menopause onset at the 13-year follow-up provides evidence of the aggravating role of cumulative resource deprivation over time in the timing of menopause. This notion is supported by prior studies in the UK^46^ and US,^47^ which found that women with lifelong exposure to socioeconomic disadvantage or economic distress exhibited earlier menopause. Additionally, our findings regarding the associations with socioeconomic, household composition, and racial and ethnic minority status subdomains of the SVI are consistent with prior studies that reported associations of the same social factors, albeit at the individual level, with age of menopause. Specifically, 2 large cohort studies of women from the US^48^ and Canada^49^ found that lower educational level and unemployment were associated with earlier age of menopause. Other studies have shown that relationship factors, such as being unmarried or having no partner, were associated with earlier menopause onset.^49,50,51^ Prior studies have also reported higher prevalence of premature menopause among Black and Hispanic women compared with White women.^52,53^ Additional research is needed to further distinguish the associations of other neighborhood-level vs individual-level social risk factors with reproductive aging outcomes.

Although SVI was associated with age of menopause onset, it was not associated with severity of menopausal symptoms. While related, menopausal timing and symptom severity represent distinct facets of reproductive aging with different underlying mechanisms and implications for future health.^7,8^ The null findings for menopausal symptoms align with results of a previous cross-sectional study that reported no association of MRS scores with socioeconomic status and occupation.^54^ Other studies, however, have reported associations of socioeconomic disadvantage at both the neighborhood^55^ and individual levels^56,57^ with greater menopausal symptom severity. This discrepancy may be partly due to different sample sizes given that other studies were larger than the present work and different methodologies used to evaluate climacteric symptoms, such as the Kupperman Index,^55,57^ which has not been validated according to psychometric standards^26,58^ and has been shown to overestimate the severity of climacteric symptoms compared with the MRS.^59^ Furthermore, previous studies^54,55,56,57^ examined only singular neighborhood indicators and thus did not elucidate the cumulative implications of multiple neighborhood stressors for menopausal symptom severity. This study directly addressed these knowledge gaps by using an integrated neighborhood index and a validated MRS.^26^

Several potential mechanisms could explain our findings. Lower socioeconomic status^60,61^ and exposure to neighborhood poverty^62^ could reduce ovarian reserve (as measured by anti-Müllerian hormone [AMH] levels, a well-established ovarian reserve marker^8^), which has been associated with earlier menopause.^63,64^ We speculate that residence in neighborhoods with very high vulnerability may lower ovarian reserve in women, potentially through stress^65^ and/or exposure to environmental pollutants,^66,67^ which in turn accelerates reproductive aging. While the study did not assess AMH levels at midlife, future research could directly explore the association between SVI and AMH levels to further elucidate the underlying pathophysiological process. Additionally, it is possible that other stressors associated with residence in disadvantaged neighborhoods, such as adverse childhood events^68^ and history of physical and sexual abuse,^14,15^ may promote earlier menopause via alterations to the sympathetic nervous system and the hypothalamic-pituitary-adrenal axis.^69,70^ Future studies in other settings could be conducted to explore these potential mechanisms.

### Strengths and Limitations

Strengths of this study include its prospective design with nearly 2 decades of follow-up, collection of high-quality data by highly trained staff using standardized protocols, and a wide range of sociodemographic covariates to minimize the implications of confounding and residential self-selection for the findings. Despite these strengths, the study has its limitations.

First, we used residential Census tracts as a marker of exposure, which have limited precision for individual addresses and may not capture the most relevant areas occupied by women. Second, the SVI does not consider other aspects of the neighborhood environment, such as crime rate and residential air quality, that have been associated with health outcomes in women, thus subjecting the findings to residual confounding. Third, the study population was restricted to parous women and those potentially undergoing natural menopausal transition; thus, the results may not be generalizable to nulligravid women or those younger than 45 years undergoing perimenopause. Fourth, differences between participants included vs excluded from this study might have contributed to some degree of selection bias. However, the analyses controlled for this issue to a certain degree through adjustment for sociodemographic factors related to selection bias^71^ and through inverse probability of censoring weighting analyses. Fifth, all participants had health insurance at enrollment, were recruited from eastern Massachusetts, and were mostly White and well-educated with high household incomes. These factors may also limit the generalizability of the findings to other populations.

## Conclusions

In this cohort study, residence in neighborhoods with very high vulnerability particularly within 10 years of the perimenopause period was associated with higher risk of earlier age of natural menopause. The study highlighted the need to address disparate contexts within neighborhoods to achieve more equitable reproductive health outcomes in women. Certain features of disadvantaged neighborhoods, such as lack of access to social and economic resources, are amenable to change through community-initiated interventions^72,73^ or policies at the local and/or federal level.^74^ Future studies are thus warranted to examine whether such strategies could mitigate the association of neighborhood disadvantage with early menopause.

## Notes

Supplement 1. **eMethods.** Secondary Analyses **eResults.** Secondary Analyses **eTable 1.** Social Vulnerability Index Indicators, Definitions, and Data Sources **eTable 2.** Characteristics of Study Participants at 8-Year and 13-Year Follow-up **eTable 3.** Characteristics of Included vs Excluded Participants **eTable 4.** Secondary Analyses for the Association of Social Vulnerability Index With Timing to Natural Menopause Onset **eFigure 1.** Distribution of Social Vulnerability Index Categories at Enrollment, 8-Years, and 13-Years Follow-up **eFigure 2.** Association of Socioeconomic SVI Subdomain at Enrollment (A), 8-Year (B), and 13-Year Follow-up (C) With Age of Natural Menopause Onset **eFigure 3.** Association of Household Composition SVI Subdomain at Enrollment (A), 8-Year (B), and 13-Year Follow-up (C) With Age of Natural Menopause Onset **eFigure 4.** Association of Racial and Ethnic Minority Status SVI Subdomain at Enrollment (A), 8-Year (B), and 13-Year Follow-up (C) With Age of Natural Menopause Onset **eFigure 5.** Association of Housing and Transportation SVI Subdomain at Enrollment (A), 8-Year (B), and 13-Year Follow-up (C) With Age of Natural Menopause Onset

Supplement 2.
Data Sharing Statement
