# Supplementary material for: Neighborhood Vulnerability and Age of Natural Menopause and Menopausal Symptoms Among Midlife Women
Source: JAMA Netw Open. 2025 May 22;8(5):e2512075. doi: 10.1001/jamanetworkopen.2025.12075 (PMC12100450; doi:10.1001/jamanetworkopen.2025.12075)
Supplement: Supplement 1. — eMethods. Secondary Analyses eResults. Secondary Analyses eTable 1. Social Vulnerability Index Indicators, Definitions, and Data Sources eTable 2. Characteristics of Study Participants at 8-Year and 13-Year Follow-up eTable 3. Characteristics of Included vs Excluded Participants eTable 4. Secondary Analyses for the Association of Social Vulnerability Index With Timing to Natural Menopause Onset eFigure 1. Distribution of Social Vulnerability Index Categories at Enrollment, 8-Years, and 13-Years Follow-up eFigure 2. Association of Socioeconomic SVI Subdomain at Enrollment (A), 8-Year (B), and 13-Year Follow-up (C) With Age of Natural Menopause Onset eFigure 3. Association of Household Composition SVI Subdomain at Enrollment (A), 8-Year (B), and 13-Year Follow-up (C) With Age of Natural Menopause Onset eFigure 4. Association of Racial and Ethnic Minority Status SVI Subdomain at Enrollment (A), 8-Year (B), and 13-Year Follow-up (C) With Age of Natural Menopause Onset eFigure 5. Association of Housing and Transportation SVI Subdomain at Enrollment (A), 8-Year (B), and 13-Year Follow-up (C) With Age of Natural Menopause Onset [file jamanetwopen-e2512075-s001.pdf]

Supplemental Online Content

Lin Z, Rifas-Shiman SL, Perng W, et al. Neighborhood vulnerability and age of natural menopause and menopausal symptoms among midlife women. *JAMA Netw Open*. 2025;8(5):e2512075. doi:10.1001/jamanetworkopen.2025.12075

eMethods. Secondary Analyses

eResults. Secondary Analyses

eTable 1. Social Vulnerability Index Indicators, Definitions, and Data Sources

eTable 2. Characteristics of Study Participants at 8-Year and 13-Year Follow-up

eTable 3. Characteristics of Included vs. Excluded Participants

eTable 4. Secondary Analyses for the Association of Social Vulnerability Index With Timing to Natural Menopause Onset

eFigure 1. Distribution of Social Vulnerability Index Categories at Enrollment, 8-Years, and 13-Years Follow-up

eFigure 2. Association of Socioeconomic SVI Subdomain at Enrollment (A), 8-Year (B), and 13-Year Follow-up (C) With Age of Natural Menopause Onset

eFigure 3. Association of Household Composition SVI Subdomain at Enrollment (A), 8-Year (B), and 13-Year Follow-up (C) With Age of Natural Menopause Onset

eFigure 4. Association of Racial and Ethnic Minority Status SVI Subdomain at Enrollment (A), 8-Year (B), and 13-Year Follow-up (C) With Age of Natural Menopause Onset

eFigure 5. Association of Housing and Transportation SVI Subdomain at Enrollment (A), 8-Year (B), and 13-Year Follow-up (C) With Age of Natural Menopause Onset

This supplemental material has been provided by the authors to give readers additional information about their work.

© 2025 Lin Z et al. *JAMA Network Open*

eMethods

Secondary analyses

We conducted several secondary analyses to assess the robustness of the findings from our main analyses. First, in a separate model, we additionally adjusted for race and ethnicity to explore whether their inclusion would meaningfully change effect estimates. Second, we additionally adjusted for Social Vulnerability Index (SVI) in all previous visits, but not in subsequent visits, to account for potential confounding from changes in SVI over time. For example, when examining the association of SVI at 8-year follow-up with menopause outcomes, we also adjusted for SVI at enrollment, but reported and interpreted the estimates of SVI at 8-year follow-up only. Third, we repeated all analyses using domain-specific rather than overall SVI categories to gain insights into specific facets of neighborhoods that may have been associated with age of menopause and menopausal symptoms.

Finally, we conducted inverse probability of censoring weighting analyses to control for potential selection bias from loss to follow-up. Briefly, we estimated stabilized censoring weights for each participant ($W^{\delta}=\frac{P(\delta=0|A)}{P(\delta=0|A, L)}$), where the denominator is the probability of being uncensored [i.e., $P(\delta=0)$] conditional on exposure *A* (i.e., SVI) and all covariates *L*, and the numerator is the probability of being uncensored conditional on the exposure. We estimated the numerator and denominator of the censoring weights using logistic regression. We then used these weights to fit weighted versions of linear regression models for association of SVI with continuous MRS scores, and Cox regression models for association of SVI with time to natural menopause onset in uncensored women. This approach generates a pseudo-population that reflects what would have been observed if the loss to follow-up had occurred randomly, without being influenced by the measured factors and baseline characteristics associated with loss to follow-up.

© 2025 Lin Z et al. *JAMA Network Open*

eResults

Secondary analyses

Results from additional adjustment of race and ethnicity for the associations of SVI at enrollment, 8-year, or 13-year follow-up with age of natural menopause were similar to our main analyses (**Model 2, eTable 4**). The association of SVI at 8-year follow-up with age of natural menopause did not change after adjusting for SVI at enrollment (**Model 3, eTable 4**). However, adjustment for SVI at both enrollment and 8-year follow-up attenuated the association of SVI at 13-year follow-up with age of natural menopause to non-significance (HR 1.21; 95% CI 0.51, 2.88). Results from inverse probability of censoring weighting analyses for the association of very high (vs. very low) SVI at enrollment, 8-, and 13-year follow-up with higher risk of earlier natural menopause onset were also similar to our main analyses (**Model 4, eTable 4**). The association of very high SVI at 8-year follow-up with earlier age of natural menopause appeared to be attributed to all 4 SVI subdomains (**eFigures 2** to **5**), while the association for very high SVI at 13-year follow-up appeared to be attributed to the socioeconomic and household composition SVI subdomains only (**eFigures 2** to **3**).

© 2025 Lin Z et al. *JAMA Network Open*

eTable 1: Social Vulnerability Index Indicators, Definitions, and Data Sources

Indicators	Definition (Data source)
Socioeconomic	
1. Poverty	Persons below poverty estimate (ACS; 2000–2018)
2. Unemployment	Civilian (age 16+) unemployed estimate (ACS; 2000–2018)
3. Income	Per capita income estimate (ACS; 2000–2018)
4. High School Diploma	Persons (age 25+) with no high school diploma estimate (ACS; 2000–2018)
Household Composition and Disability	
1. Aged 65 or Older	Persons aged 65 and older estimate (ACS; 2000–2018)
2. Aged 17 or Younger	Persons aged 17 and younger estimate (ACS; 2000–2018)
3. Civilian with a Disability	Civilian noninstitutionalized population with a disability estimate (ACS; 2000–2018)
4. Single-Parent Households	Single parent household with children under 18 estimate (ACS; 2000–2018)
Minority Status and Language	
1. Minority	Minority (all persons except white, non-Hispanic) estimate (ACS; 2000–2018)
2. Speaks English "Less than Well"	Persons (age 5+) who speak English "less than well" estimate (ACS; 2000–2018)
Housing and Transportation	
1. Multi-Unit Structures	Housing in structures with 10 or more units estimate (ACS; 2000–2018)
2. Mobile Homes	Mobile homes estimate (ACS; 2000–2018)
3. Crowding	At household level (occupied housing units), more people than rooms estimate (ACS; 2000–2018)
4. No Vehicle	Households with no vehicle available estimate (ACS; 2000–2018)
5. Group Quarters	Persons in institutionalized and noninstitutionalized group quarters estimate (ACS; 2000–2018)

ACS: American Community Survey.

eTable 2: Characteristics of study participants at 8-year and 13-year follow-up.

Characteristics at 8-year follow-up	SVI categories at 8-year follow-up						p-value
	Overall n=635	Very Low n=277	Low n=197	Moderate n=74	High n=49	Very High n=38	
**Annual household income >$70,000/y**							<0.001
. No	124 (20.3)	26 (9.7)	34 (18.0)	19 (27.5)	23 (47.9)	22 (61.1)	
. Yes	487 (79.7)	243 (90.3)	155 (82.0)	50 (72.5)	25 (52.1)	14 (38.9)	
**Employment status at 1-year**							0.24
. Unemployed	154 (27.7)	71 (27.7)	55 (31.1)	19 (28.4)	7 (20.0)	2 (9.5)	
. Employed	402 (72.3)	185 (72.3)	122 (68.9)	48 (71.6)	28 (80.0)	19 (90.5)	
**Smoking status**							0.04
. Never	457 (73.2)	196 (71.8)	143 (73.7)	53 (74.6)	36 (73.5)	29 (78.4)	
. Former	144 (23.1)	69 (25.3)	44 (22.7)	18 (25.4)	7 (14.3)	6 (16.2)	
. Current	23 (3.7)	8 (2.9)	7 (3.6)	0 (0.0)	6 (12.2)	2 (5.4)	
**No. of cigarettes smoked per day**	0.2 (1.4)	0.2 (1.2)	0.2 (1.4)	0.0 (0.0)	0.8 (3.1)	0.3 (1.6)	0.06
**BMI in kg/m^2, mean (SD)**	26.3 (5.9)	25.2 (4.8)	26.0 (5.9)	26.9 (5.8)	28.8 (6.7)	30.5 (8.5)	<0.001

Characteristics at 13-year follow-up	SVI categories at 13-year follow-up						p-value
	Overall n=660	Very Low n=315	Low n=177	Moderate n=80	High n=47	Very High n=41	
**Annual household income >$70,000/y**							<0.001
. No	120 (20.1)	26 (9.0)	26 (16.3)	26 (38.2)	21 (47.7)	21 (60.0)	
. Yes	476 (79.9)	263 (91.0)	134 (83.8)	42 (61.8)	23 (52.3)	14 (40.0)	
**Employment status**							0.25
. Unemployed	155 (27.6)	76 (27.7)	52 (31.1)	16 (25.8)	8 (21.6)	3 (14.3)	
. Employed	406 (72.4)	198 (72.3)	115 (68.9)	46 (74.2)	29 (78.4)	18 (85.7)	
**Smoking status**							0.01
. Never	440 (72.4)	207 (70.9)	117 (71.3)	55 (76.4)	33 (75.0)	28 (77.8)	
. Former	145 (23.8)	76 (26.0)	42 (25.6)	15 (20.8)	6 (13.6)	6 (16.7)	
. Current	23 (3.8)	9 (3.1)	5 (3.0)	2 (2.8)	5 (11.4)	2 (5.6)	
**No. of cigarettes smoked per day**	0.2 (1.5)	0.2 (1.3)	0.2 (1.5)	0.0 (0.3)	0.6 (3.0)	0.3 (1.6)	0.58
**BMI in kg/m^2, mean (SD)**	26.3 (5.9)	25.4 (5.2)	25.9 (5.3)	26.7 (6.6)	29.0 (6.8)	30.1 (8.4)	<0.001

© 2025 Lin Z et al. *JAMA Network Open*

eTable 3: Characteristics of included vs. excluded participants.

**Characteristic**	**Included** n=691	**Excluded** n=1409	**p-value**
	**Mean (SD) or N (%)**		
**Age at enrollment, years**	33.7 (3.8)	30.9 (5.6)	<0.001
**Annual household income >$70,000/y**			<0.001
. No	206 (32)	519 (43)	
. Yes	443 (68)	681 (57)	
**College education**			<0.001
. No	139 (20)	597 (43)	
. Yes	550 (80)	790 (57)	
**Employment status**			0.04
. Unemployed	80 (12)	180 (16)	
. Employed	564 (88)	938 (84)	
**Pregnancy smoking status**			<0.001
. Never	498 (72)	927 (67)	
. Former	137 (20)	252 (18)	
. Smoked during pregnancy	54 (8)	211 (15)	
**No. of cigarettes smoked per day**	0.2 (0.9)	0.2 (1.0)	0.054
**Race and ethnicity**			<0.001
. Non-Hispanic Asian	41 (6)	77 (6)	
. Non-Hispanic Black	79 (11)	267 (19)	
. Hispanic	39 (6)	113 (8)	
. Non-Hispanic White	507 (74)	872 (63)	
. Non-Hispanic Other[a]	23 (3)	58 (4)	
**Pre-pregnancy body mass index, kg/m^2**	24.5 (5.1)	25.1 (5.7)	0.02
**Overall SVI category at enrollment**			<0.001
. Very Low Vulnerability	240 (35)	389 (28)	
. Low Vulnerability	145 (21)	281 (20)	
. Moderate Vulnerability	142 (21)	300 (21)	
. High Vulnerability	69 (10)	148 (11)	
. Very High Vulnerability	87 (13)	280 (20)	

[a] Non-Hispanic Other race categories include American Indian, Alaskan Native, Pacific Islander, or Other race (unspecified).

© 2025 Lin Z et al. *JAMA Network Open*

eTable 4: Secondary analyses for the association of Social Vulnerability Index with timing to natural menopause onset.

	Hazard Ratio (95% CI)			
	**Model 1**	**Model 2**	**Model 3**	**Model 4**
**SVI at enrollment[a]**				
. Very Low Vulnerability	ref	ref	–	ref
. Low Vulnerability	1.19 (0.87, 1.63)	1.20 (0.88, 1.65)	–	1.13 (0.80, 1.60)
. Moderate Vulnerability	1.25 (0.90, 1.73)	1.25 (0.90, 1.74)	–	1.18 (0.82, 1.70)
. High Vulnerability	1.48 (0.97, 2.26)	1.48 (0.96, 2.28)	–	1.43 (0.87, 2.37)
. Very High Vulnerability	1.36 (0.90, 2.06)	1.35 (0.86, 2.13)	–	1.25 (0.72, 2.15)
**SVI at 8y follow-up[b]**				
. Very Low Vulnerability	ref	ref	ref	ref
. Low Vulnerability	1.03 (0.78, 1.36)	1.04 (0.78, 1.38)	1.02 (0.76, 1.35)	1.03 (0.76, 1.39)
. Moderate Vulnerability	1.15 (0.76, 1.74)	1.19 (0.78, 1.81)	1.14 (0.73, 1.77)	1.09 (0.69, 1.73)
. High Vulnerability	1.56 (0.96, 2.53)	1.60 (0.97, 2.64)	1.54 (0.89, 2.69)	1.73 (1.00, 3.01)
. Very High Vulnerability	**2.23 (1.29, 3.85)**	**2.32 (1.20, 4.48)**	**2.21 (1.19, 4.11)**	2.17 (0.99, 4.74)
**SVI at 13y follow-up[c]**				
. Very Low Vulnerability	ref	ref	ref	ref
. Low Vulnerability	1.06 (0.80, 1.41)	1.07 (0.80, 1.42)	1.02 (0.74, 1.39)	1.07 (0.79, 1.46)
. Moderate Vulnerability	0.78 (0.52, 1.18)	0.78 (0.51, 1.19)	0.67 (0.40, 1.10)	0.77 (0.48, 1.23)
. High Vulnerability	1.07 (0.65, 1.74)	1.11 (0.66, 1.85)	0.74 (0.37, 1.46)	1.17 (0.66, 2.08)
. Very High Vulnerability	**2.18 (1.30, 3.66)**	**2.31 (1.16, 4.59)**	1.21 (0.51, 2.88)	**2.38 (1.11, 5.11)**

Bolded values reflect p<0.05.
Model 1[a]: Adjusted for covariates at enrollment (i.e., age, education level, annual household income, employment status, smoking status, number of cigarettes smoked per day, and pre-pregnancy body mass index).
Model 1[b]: Adjusted for covariates at enrollment (i.e., age, education level, annual household income, smoking status, and pre-pregnancy body mass index), 1-year (i.e., employment status), and 8-year follow-up (i.e., annual household income, smoking status, and number of cigarettes smoked per day, and body mass index).
Model 1[c]: Adjusted for covariates at enrollment (i.e., age, education level, annual household income, smoking status, and pre-pregnancy body mass index) and 13-year follow-up (i.e., annual household income, employment status, smoking status, number of cigarettes smoked per day, and body mass index).
Model 2: Model 1 + race and ethnicity.
Model 3: Model 1 + SVI at prior timepoint(s).
Model 4: Inverse probability of censoring weighting analyses to control for potential selection bias from loss to follow-up.

© 2025 Lin Z et al. *JAMA Network Open*

eFigure 1: Distribution of Social Vulnerability Index categories at enrollment, 8-years, and 13-years follow-up.

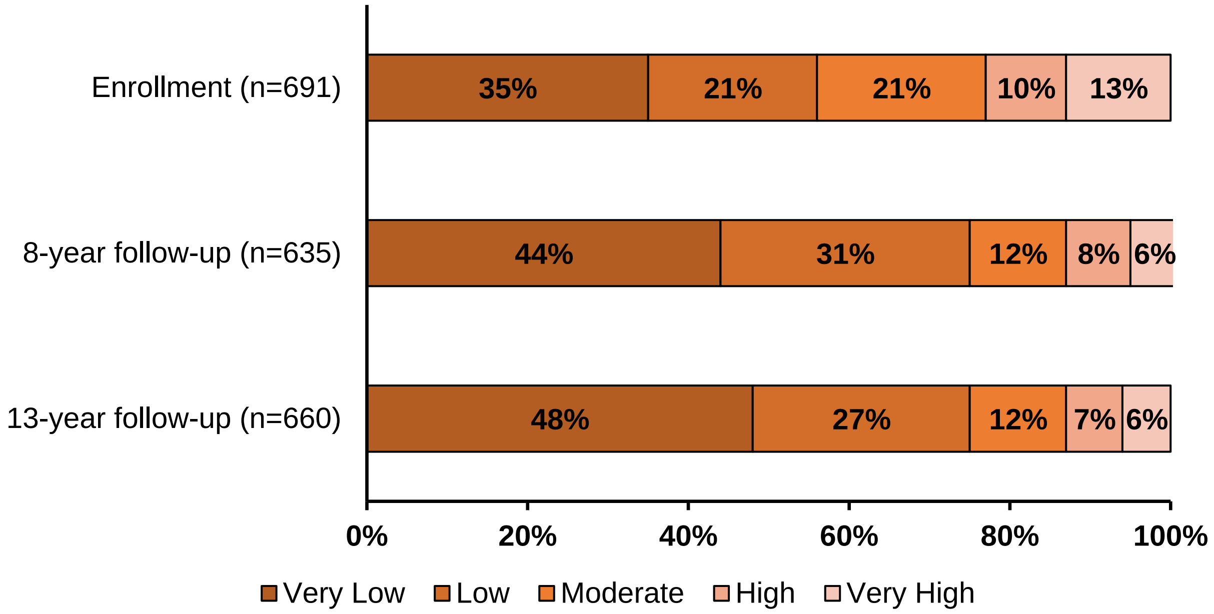

© 2025 Lin Z et al. *JAMA Network Open*

eFigure 2: Association of socioeconomic SVI subdomain at enrollment (**A**), 8-year (**B**), and 13-year follow-up (**C**) with age of natural menopause onset.

Estimates for SVI at enrollment were adjusted for covariates at enrollment (i.e., age, education level, annual household income, smoking status, and pre-pregnancy body mass index). Estimates for SVI at 8-year follow-up were adjusted for covariates at enrollment (i.e., age, education level, annual household income, smoking status, and pre-pregnancy body mass index), 1-year (i.e., employment status), and 8-year follow-up (i.e., annual household income, smoking status, number of cigarettes smoked per day, and body mass index). Estimates for SVI at 13-year follow-up were adjusted for covariates at enrollment (i.e., age, education level, annual household income, smoking status, and pre-pregnancy body mass index) and 13-year follow-up (i.e., employment status, annual household income, smoking status, number of cigarettes smoked per day, and body mass index).

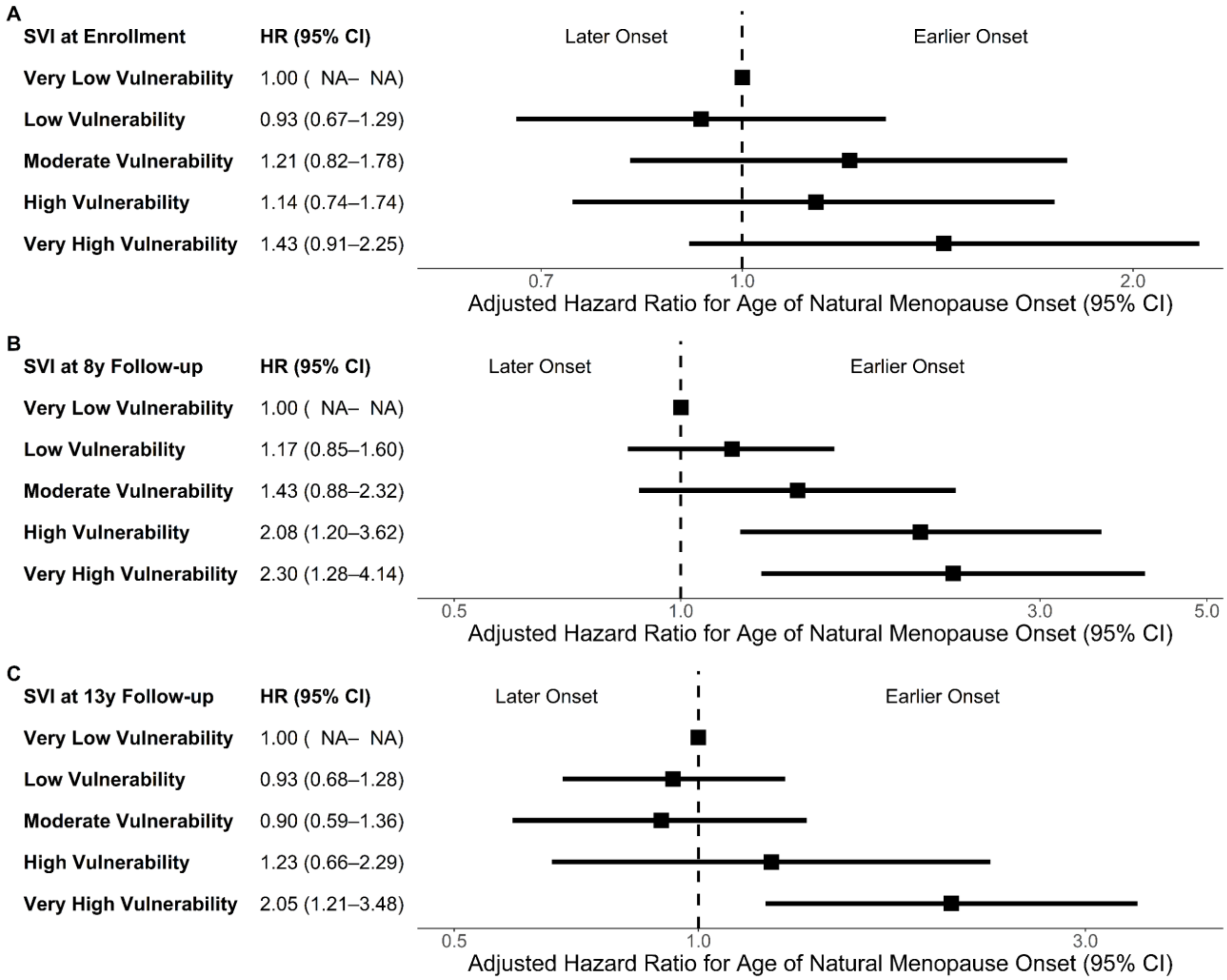

© 2025 Lin Z et al. *JAMA Network Open*

eFigure 3: Association of household composition SVI subdomain at enrollment (**A**), 8-year (**B**), and 13-year follow-up (**C**) with age of natural menopause onset.

Estimates for SVI at enrollment were adjusted for covariates at enrollment (i.e., age, education level, annual household income, smoking status, and pre-pregnancy body mass index). Estimates for SVI at 8-year follow-up were adjusted for covariates at enrollment (i.e., age, education level, annual household income, smoking status, and pre-pregnancy body mass index), 1-year (i.e., employment status), and 8-year follow-up (i.e., annual household income, smoking status, number of cigarettes smoked per day, and body mass index). Estimates for SVI at 13-year follow-up were adjusted for covariates at enrollment (i.e., age, education level, annual household income, smoking status, and pre-pregnancy body mass index) and 13-year follow-up (i.e., employment status, annual household income, smoking status, number of cigarettes smoked per day, and body mass index).

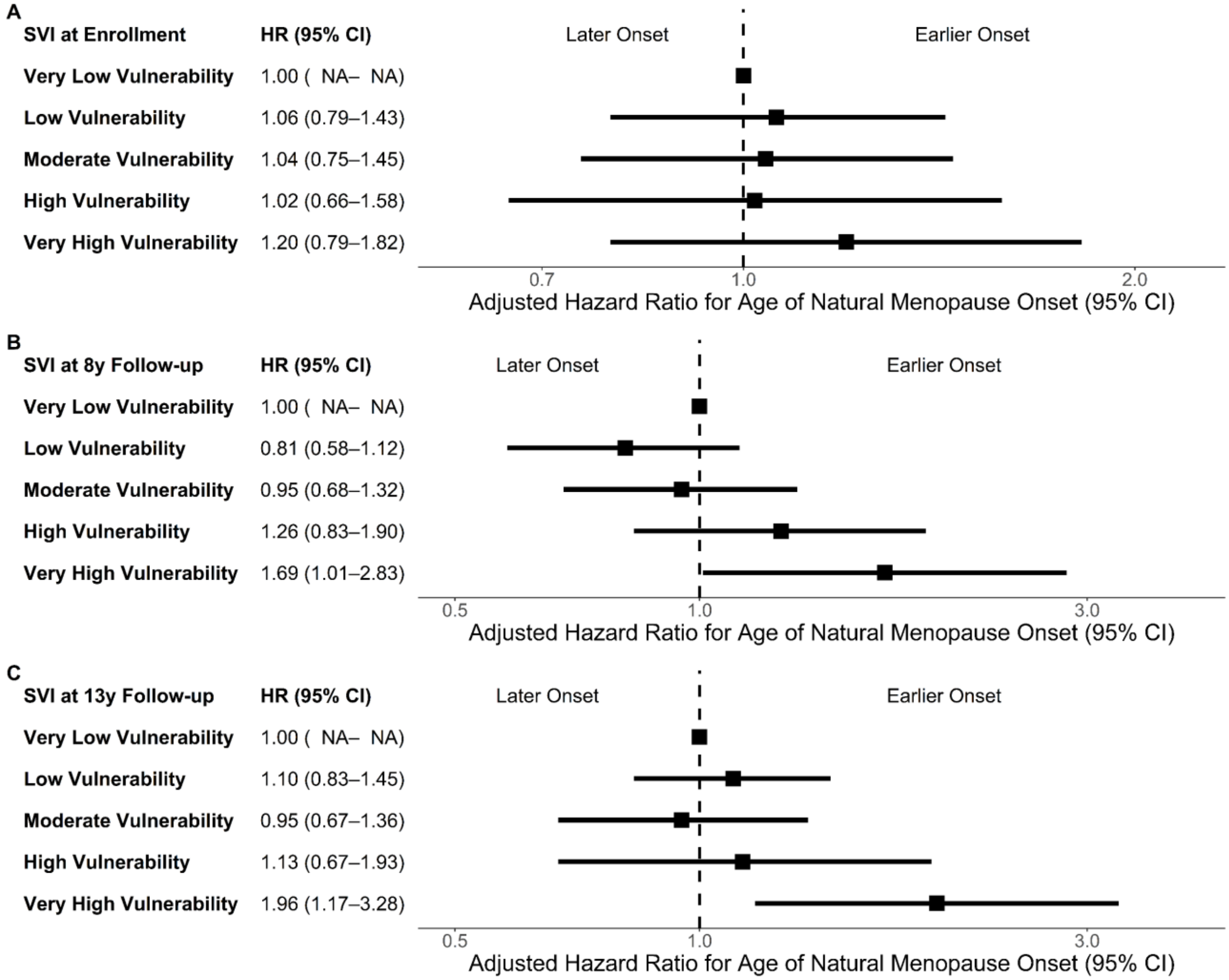

© 2025 Lin Z et al. *JAMA Network Open*

eFigure 4: Association of racial and ethnic minority status SVI subdomain at enrollment (**A**), 8-year (**B**), and 13-year follow-up (**C**) with age of natural menopause onset.

Estimates for SVI at enrollment were adjusted for covariates at enrollment (i.e., age, education level, annual household income, smoking status, and pre-pregnancy body mass index). Estimates for SVI at 8-year follow-up were adjusted for covariates at enrollment (i.e., age, education level, annual household income, smoking status, and pre-pregnancy body mass index), 1-year (i.e., employment status), and 8-year follow-up (i.e., annual household income, smoking status, number of cigarettes smoked per day, and body mass index). Estimates for SVI at 13-year follow-up were adjusted for covariates at enrollment (i.e., age, education level, annual household income, smoking status, and pre-pregnancy body mass index) and 13-year follow-up (i.e., employment status, annual household income, smoking status, number of cigarettes smoked per day, and body mass index).

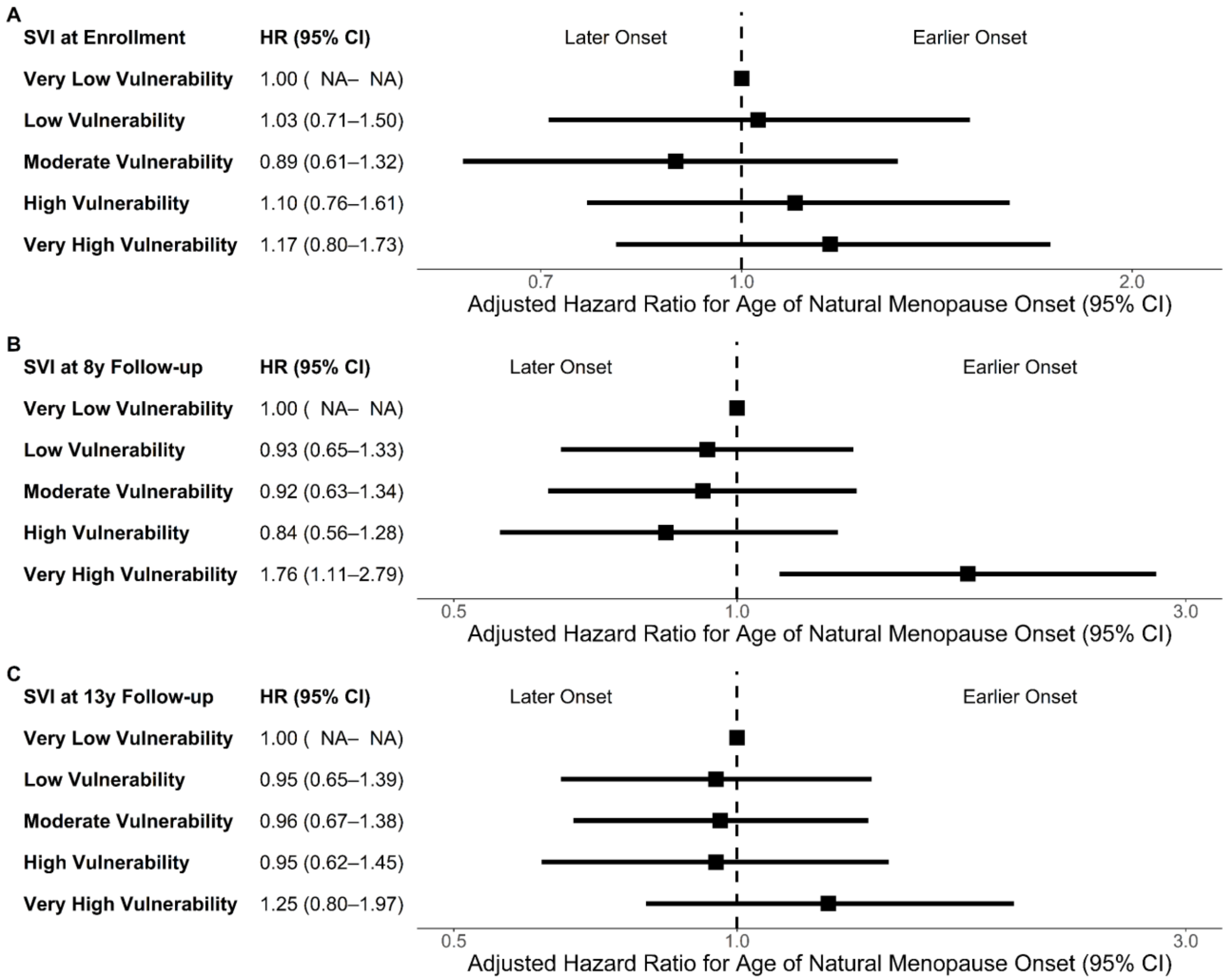

© 2025 Lin Z et al. *JAMA Network Open*

eFigure 5: Association of housing and transportation SVI subdomain at enrollment (**A**), 8-year (**B**), and 13-year follow-up (**C**) with age of natural menopause onset.

Estimates for SVI at enrollment were adjusted for covariates at enrollment (i.e., age, education level, annual household income, smoking status, and pre-pregnancy body mass index). Estimates for SVI at 8-year follow-up were adjusted for covariates at enrollment (i.e., age, education level, annual household income, smoking status, and pre-pregnancy body mass index), 1-year (i.e., employment status), and 8-year follow-up (i.e., annual household income, smoking status, number of cigarettes smoked per day, and body mass index). Estimates for SVI at 13-year follow-up were adjusted for covariates at enrollment (i.e., age, education level, annual household income, smoking status, and pre-pregnancy body mass index) and 13-year follow-up (i.e., employment status, annual household income, smoking status, number of cigarettes smoked per day, and body mass index).

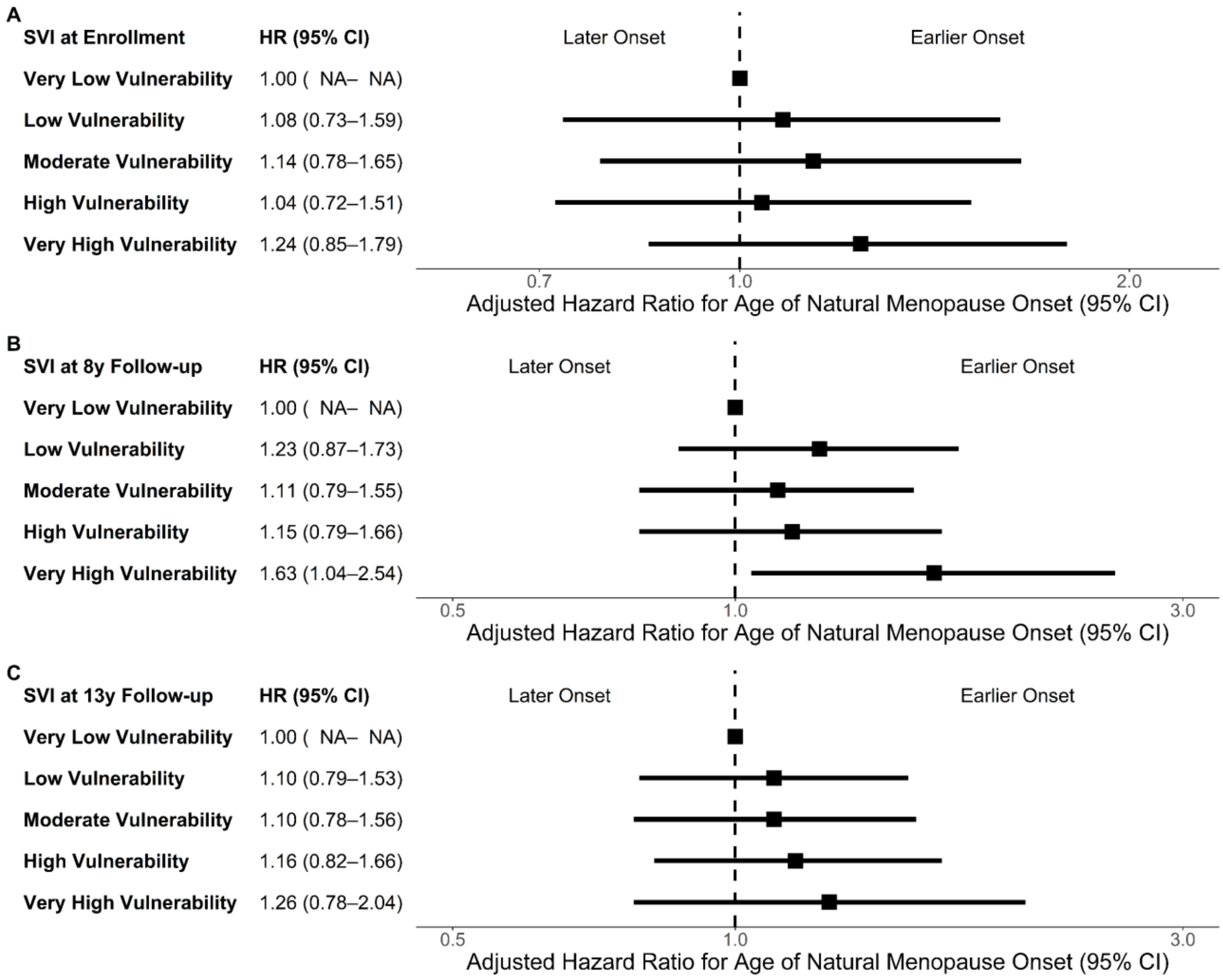

© 2025 Lin Z et al. *JAMA Network Open*
