# Supplementary material for: Neighborhood Vulnerability and Age of Natural Menopause and Menopausal Symptoms Among Midlife Women
Source: JAMA Netw Open. 2025 May 22;8(5):e2512075. doi: 10.1001/jamanetworkopen.2025.12075 (PMC12100450; doi:10.1001/jamanetworkopen.2025.12075)
Supplement: Supplement 2. — Data Sharing Statement [file jamanetwopen-e2512075-s002.pdf]

Data Sharing Statement

Lin. Neighborhood Vulnerability and Age of Natural Menopause and Menopausal Symptoms Among Midlife Women. *JAMA Netw Open*. Published May 22, 2025.
doi:10.1001/jamanetworkopen.2025.12075

Data
Data available: Yes
Data types: Deidentified participant data
How to access data: Data described in the article will be made available upon request pending application to the following email: VivaROADMaP@hphci.harva
When available: With publication

Supporting Documents
Document types: Statistical/analytic code
How to access documents: Code book and analytic code will be made available upon request pending application to the following email: VivaROADMaP@hphci.harvard.edu
When available: With publication

Additional Information
Who can access the data: Data will be made available to researchers whose proposed use of the data has been approved.
Types of analyses: For a specified purpose
Mechanisms of data availability: After approval of an analytic proposal and a signed data access agreement.
